# Supplementary figures and images for: Estimating information in time-varying signals
Source: PLoS Comput Biol. 2019 Sep 3;15(9):e1007290. doi: 10.1371/journal.pcbi.1007290 (PMC6743786; doi:10.1371/journal.pcbi.1007290)

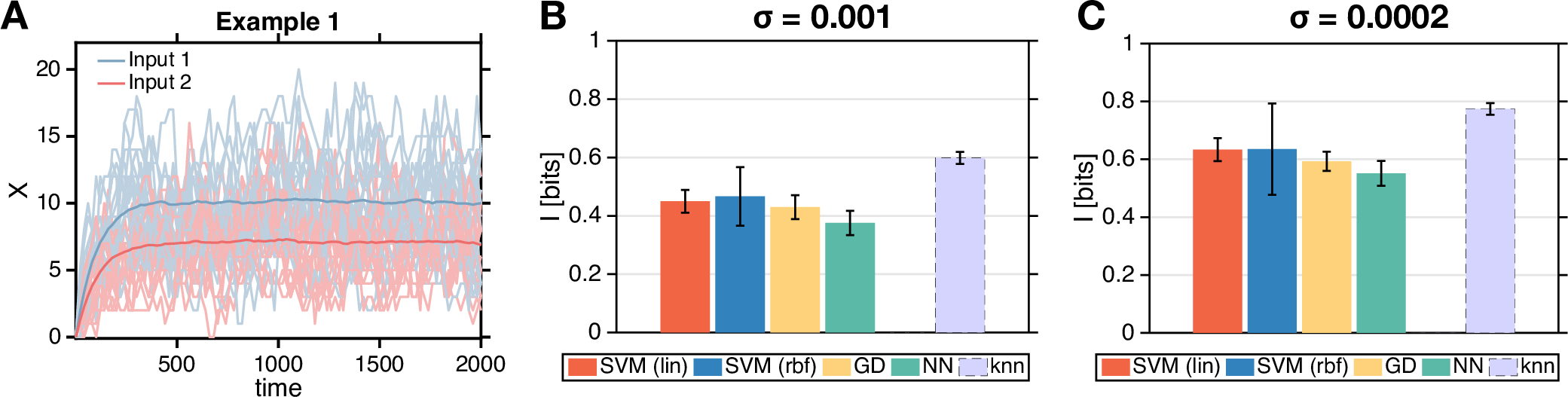

Supplement: S1 Fig — (A) Sample trajectories as in Example 1 of Fig 2A, using the same parameters and plotting conventions. Extrinsic noise was introduced by perturbing the degradation rate (fixed at 0.01 for all cells in the main paper), by adding a Gaussian IID distributed random variable with σ = 0.001 (shown in A and B) or σ = 0.0002 (shown in C); the random variable is drawn separately for each cell at the beginning of the simulation and is held fixed through time. (B,C) Estimator performance on test data (where extrinsic noise is also resampled for each cell in the test set) for both extrinsic noise levels. SVM and knn estimators perform best, but unlike in the main paper, here we do not have a reference comparison of the MAP decoder. While decoding-based estimators are giving a conservative lower-bound, we have no guarantees of whether knn extracts more information (and thus has better performance), or actually overestimates decodable information. (TIF) [file pcbi.1007290.s001.tif]

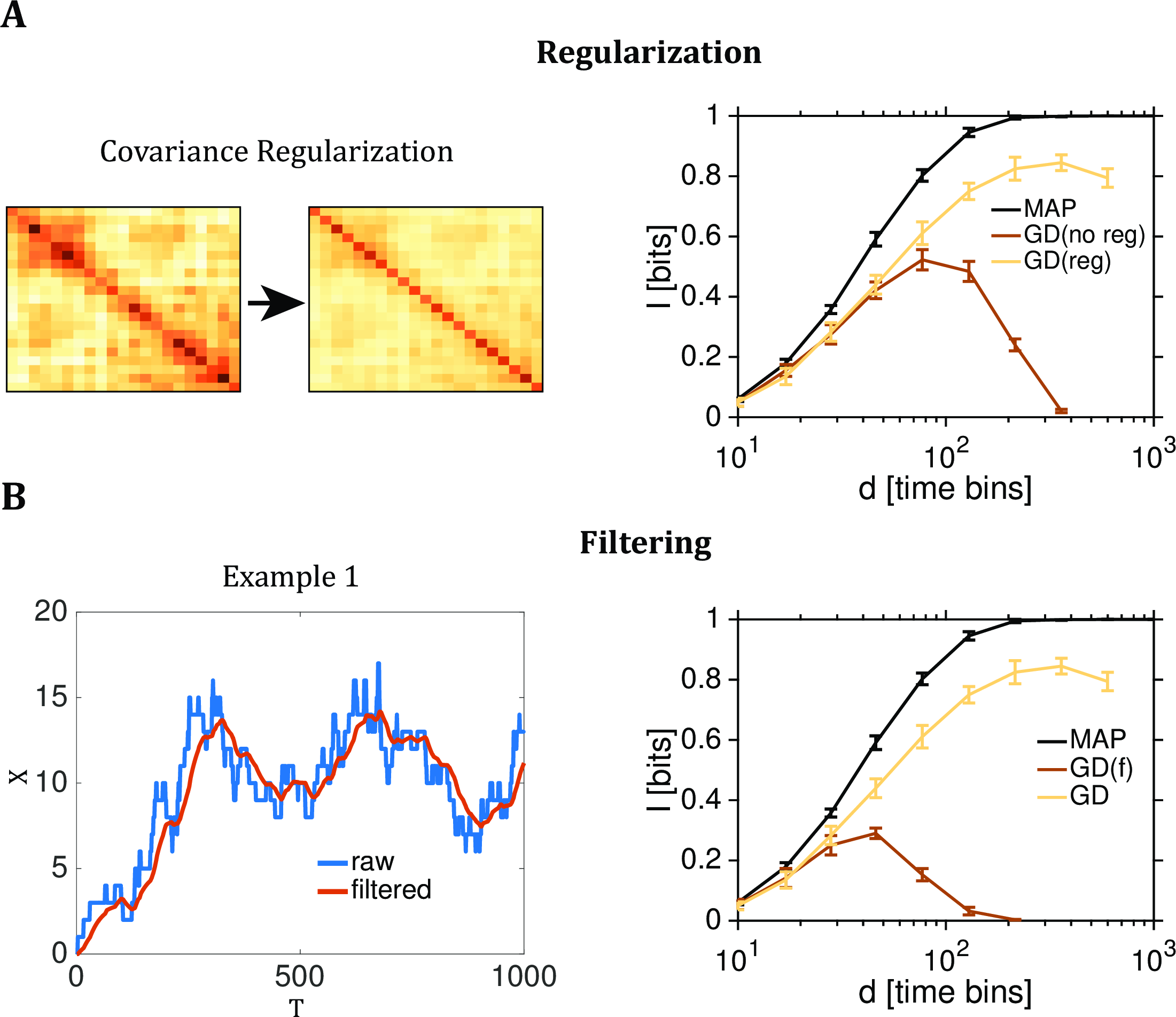

Supplement: S2 Fig — (A) At left. Diagonal covariance regularization following Ref 64 of the main paper. Briefly, λ times the identity matrix is added to the empirical covariance matrix with the hyperparameter λ set so that the likelihood on test data is maximized. Shown is the empirical (left) and regularized (right) covariance matrix for Example 3, using d = 20 and N = 30 sample trajectories. At right. Information estimates for Example 3: IMAP decoding bound (black), Gaussian decoder estimate, IGD(reg), with optimal diagonal regularization for each d (yellow, as in Fig 5C), Gaussian decoder estimate, IGD(noreg) (brown). Without regularization, the estimate suffers an abrupt drop as d increases and the empirically estimated covariance matrix becomes close to singular. N and plotting conventions are as in Fig 5. (B) The effects of trajectory filtering on information estimates. At left. A raw integer-valued stochastic trajectory for X˜ (blue) can be filtered by a low-pass exponential decay filter with adjustable timescale, τ = 1 − 103, here τ = 50 (red) to yield real-valued trajectory. At right. Regularized Gaussian-decoder information estimates with (brown) and without (yellow) filtering. Filtering does not improve but can decrease the estimation performance, even when the filtering timescale is adjusted. (TIF) [file pcbi.1007290.s002.tif]

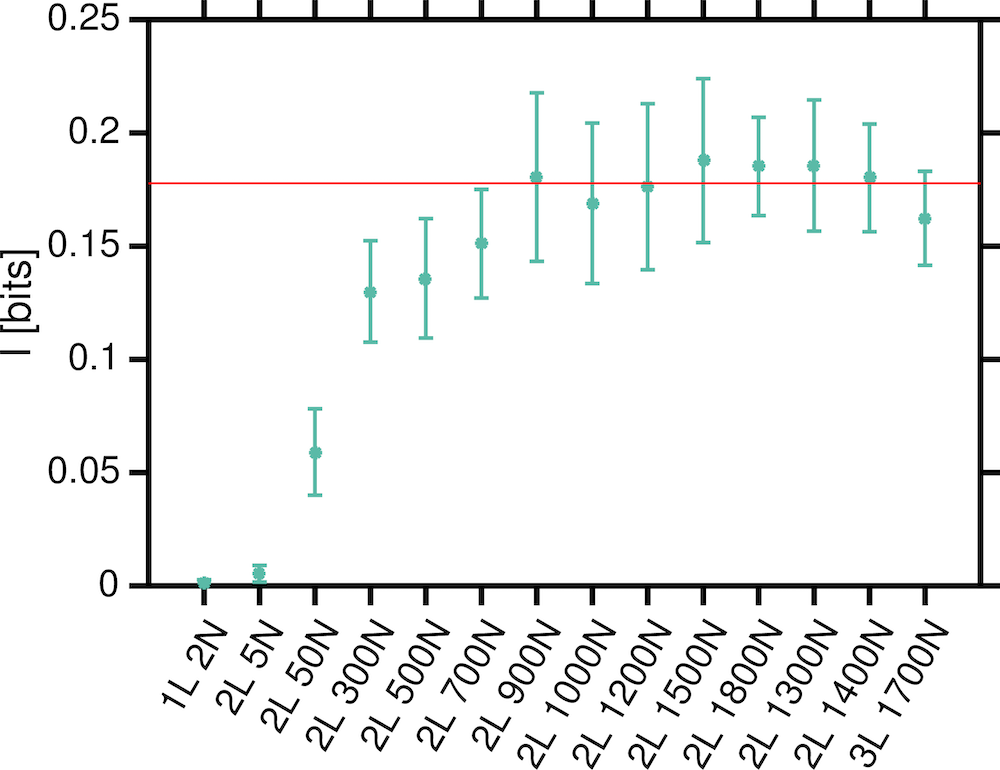

Supplement: S3 Fig — Shown are INN estimates on Example 3, analogous to Fig 5C and 5F of the main paper. Decoders are trained on N = 1000 sample trajectories per input condition, and trajectories are represented as d = 50 dimensional vectors. The performance of the network architecture used in the main paper is shown as the horizontal red line. Alternative architectures are denoted on the x-axis label. “1L, 2L, 3L” stands for 1, 2 or 3 layers, respectively. The number in front of letter “N” represents the total number of neurons on the hidden layers. Other technical details of the networks as reported in the Methods. (TIF) [file pcbi.1007290.s003.tif]

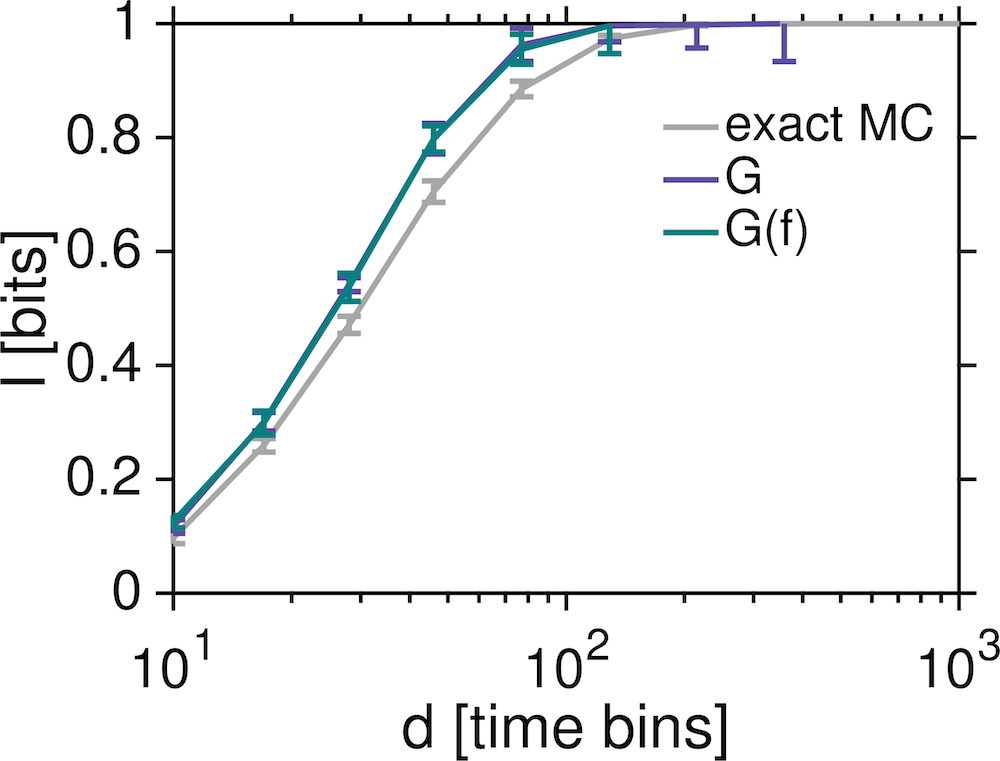

Supplement: S4 Fig — Gaussian approximation is evaluated for Example 3 in Fig 5C, using N = 1000 per condition. Exact Monte Carlo approximation of the information, Iexact(X; U), is shown in dark gray. Information estimates following Section Model-free information estimators are shown in violet (Gaussian approximation for raw, integer-valued response trajectories) or in cyan (Gaussian approximation for filtered trajectories), as in S2 Fig. In both cases the Gaussian approximation overshoots the true information value. Further numerical analyses indicated that the difference is hard to predict and that it persists even when the reaction rates are chosen such that the mean expression level is ten-fold higher (and the intrinsic stochasticity correspondingly lower). This makes direct Gaussian approximation risky to use, in contrast to the Gaussian-decoder based estimate, which is guaranteed to stay below Iexact. (TIF) [file pcbi.1007290.s004.tif]

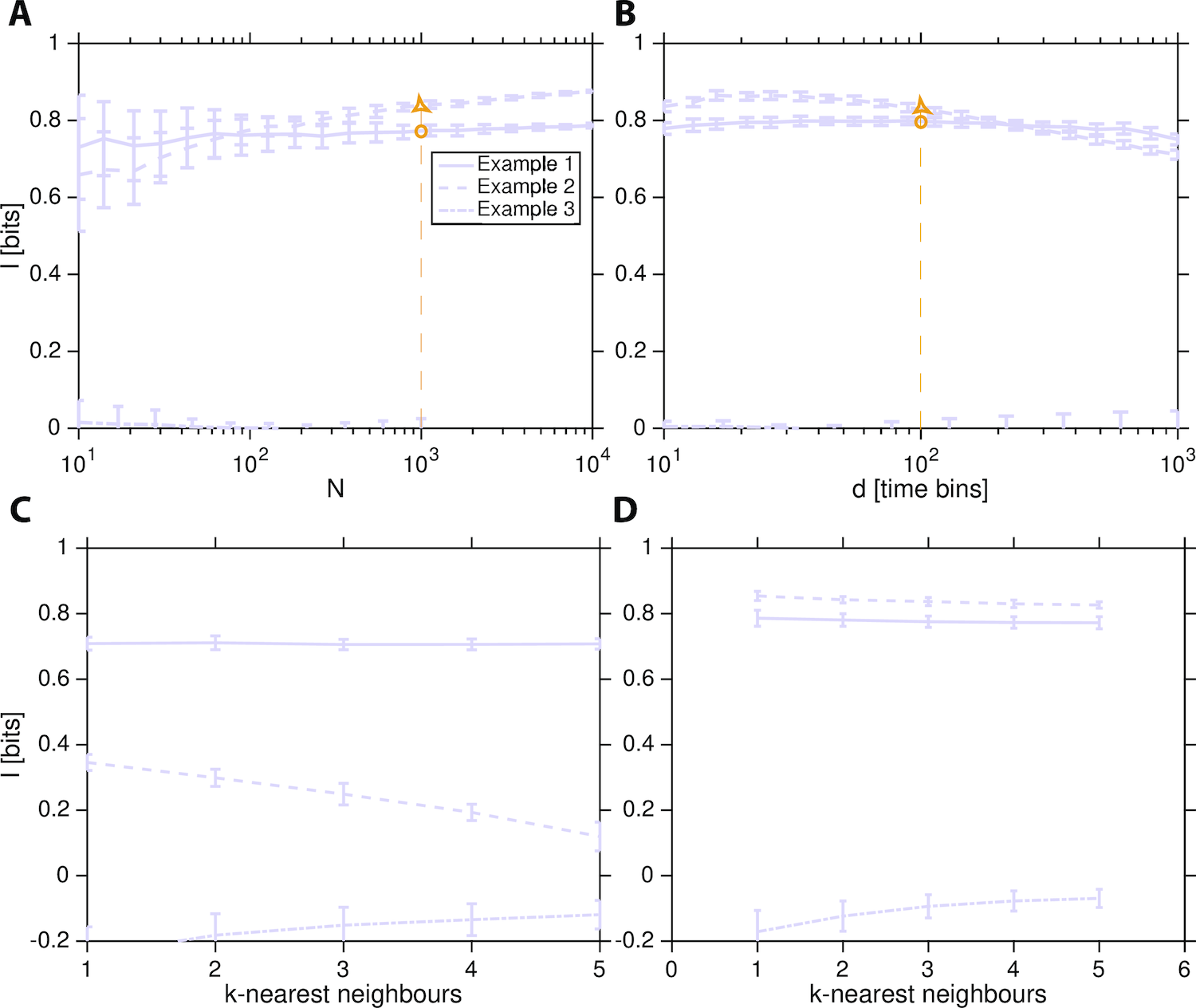

Supplement: S5 Fig — Compared to knn results in Fig 7, the results in A, B and D are estimated following the same procedure, while adding a small amount of IID zero-mean Gaussian noise to each response trajectory at every time bin; the noise variance must be ≪1 but otherwise does not affect the results much. This results in good estimates even at low sample number, N, and provides nearly stable estimation as a function of the trajectory dimension, d, for Example 1 and Example 2. It, however, does not resolve the estimator failure for Example 3. (A) Dependence of the knn estimator performance on the number of samples. Yellow plot symbols indicate the number of samples per condition, N = 103, used in Fig 7. (B) Dependence of the knn estimator performance on the trajectory dimension. Yellow plot symbols indicate the dimension, d = 102, used in Fig 7. (C, D) Dependence of the knn estimates on the number of nearest neighbors, k, at N = 103 and d = 102, without the addition of noise (C) or with the addition of noise (D). (TIF) [file pcbi.1007290.s005.tif]

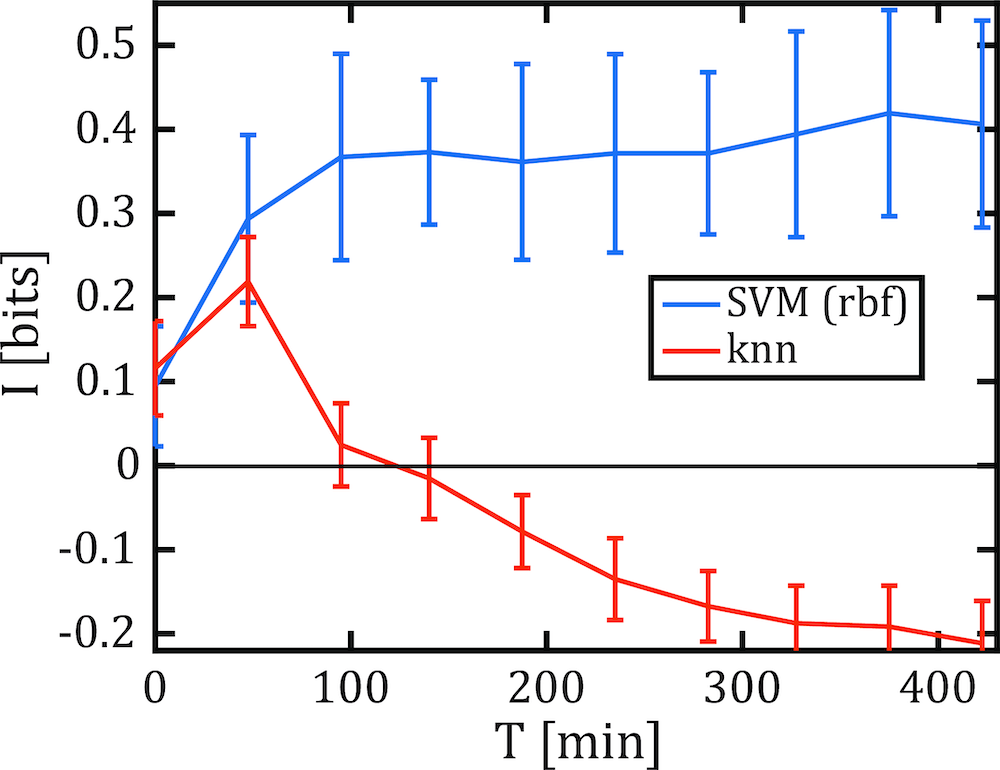

Supplement: S6 Fig — When the samples are limited, here to N = 100 samples per input glucose level condition as in Fig 8A (middle), radial-basis-function SVM estimate (blue) is well-behaved with no observable overfitting and consequent drop in information estimate as the trajectory duration, T, is increased (maximal T corresponds to d = 170 dimensional trajectory vectors). In contrast, knn estimate (brown) shows a collapse in the estimation performance, even yielding strongly negative numbers, as the dimensionality of input vectors is increased at fixed number of trajectory samples. (TIF) [file pcbi.1007290.s006.tif]

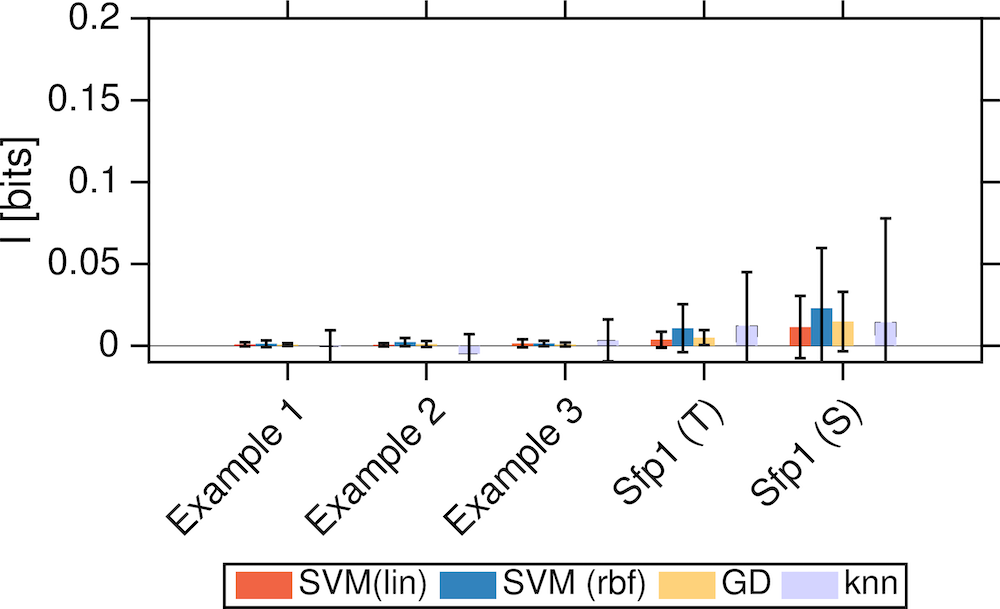

Supplement: S7 Fig — By randomly shuffling the binary labels assigned to different response trajectories, we break all response-input correlations leading to zero information. Here we test whether our estimators correctly report zero information within error bars given a finite number of samples, or are subject to positive information estimation bias. Decoding-based estimates (linear SVM, red; kernelized SVM, blue; Gaussian decoder, yellow) and knn (gray). First three sets of bars correspond to synthetic examples of Fig 3; estimations are done with d = 100 and N = 1000 per input condition as in Fig 5, following the same plotting conventions. Last two sets of bars are estimated with N = 100 per input condition using real data for Sfp1 yeast TF from Fig 8A. In all cases, even without explicit small-sample debiasing for Eq (26) (which may be required for multilevel estimation), the estimates are consistent with zero. (TIF) [file pcbi.1007290.s007.tif]

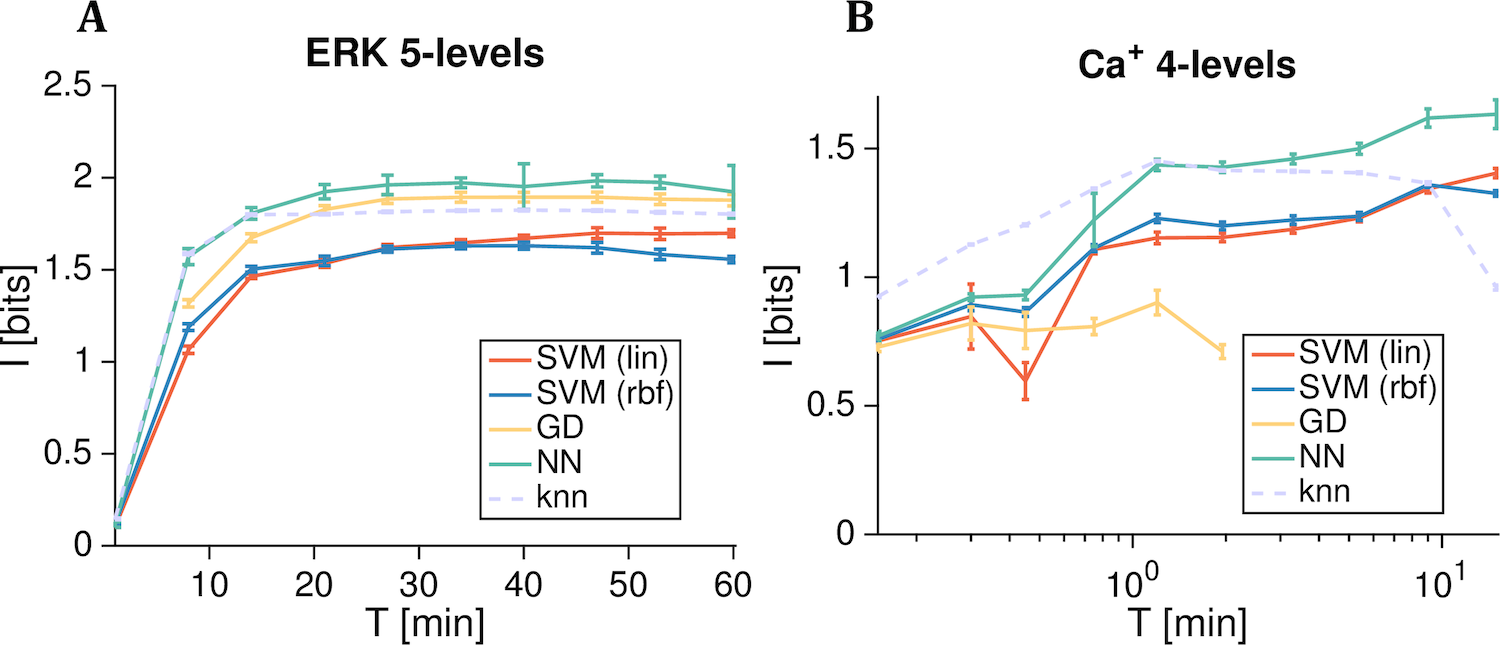

Supplement: S8 Fig — Shown are information estimates as a function of the total trajectory duration, T, for the early response period for ERK (A) and Ca2+ (B). Plotting conventions, procedures, and data set sizes same as in Fig 9. (TIF) [file pcbi.1007290.s008.tif]
